# Early Warning Indicators of Severe COVID-19: A Single-Center Study of Cases From Shanghai, China

**DOI:** 10.3389/fmed.2020.00432

**Published:** 2020-07-17

**Authors:** Yiming Lu, Kuo Sun, Shanshan Guo, Junjie Wang, An Li, Xuli Rong, Tingfang Wang, Yan Shang, Wenjun Chang, Sheng Wang

**Affiliations:** ^1^Department of Critical Care Medicine, School of Medicine, Shanghai Tenth People's Hospital, Tongji University, Shanghai, China; ^2^Department of Biochemical Pharmacy, School of Pharmacy, Second Military Medical University, Shanghai, China; ^3^School of Medicine, Shanghai University, Shanghai, China; ^4^Department of Respiratory and Critical Care Medicine, Changhai Hospital, Second Military Medical University, Shanghai, China; ^5^Department of Environment and Occupation Health, Second Military Medical University, Shanghai, China

**Keywords:** COVID-19, early warning indicators, severe patients, moderate patients, adult

## Abstract

**Background:** Patients with severe novel coronavirus disease (COVID-19) can likely develop comorbidities, which can lead to irreversible organ damage and, eventually, death. However, early indicators of disease progression remain unclear. This study aimed to identify early indicators of disease progression to provide a basis for improved prognostic prediction and disease management.

**Methods:** We examined 53 recovered adult COVID-19 patients who were treated at Shanghai Public Health Clinical Center between January 20, 2020, and February 20, 2020. The patients were categorized into the following four groups according to their condition at admission: mild condition (*n* = 3), moderate (*n* = 41), severe (*n* = 7), and critical (*n* = 2). They were also categorized according to disease progression as mild or moderate conditions that remained stable (*n* = 26), moderate disease that progressed to severe condition (*n* = 18), and continuously severe or critical (*n* = 9). We then focused on investigating the differences in the epidemiological and laboratory indicators between remained stable cases and progressed to severe condition cases.

**Results:** Mild or moderate patients were younger than severe or critical patients. The number of patients with shortness of breath and underlying diabetes and heart disease at admission was higher in the severe or critical group. This group also showed considerably lower or higher values in 28 laboratory indicators. In addition, mild and moderate patients who remained stable were younger than moderate patients progressing to severe disease. Men had a higher risk of disease progression. Patients who progressed had either higher or lower values in 11 laboratory indicators. Survival curve analysis showed that age, procalcitonin, D-dimer, serum C-reactive protein, lactate dehydrogenase, lymphocytes, neutrophils, CD4%, and CD4/CD8 ratio were significant predictors of progression to severe disease.

**Conclusions:** Lactate dehydrogenase, procalcitonin, etc. are early warning indicators of severe COVID-19. Age (>64 years), shortness of breath, past histories of diabetes and heart disease, and abnormality in 28 other indicators at admission are indicative of severe or progression toward severe COVID-19. Meanwhile, abnormalities in 11 indicators and an abnormal coagulation function index at admission are risk factors for progression to severe disease.

## Introduction

The novel coronavirus disease (COVID-19) has spread rapidly and become a pandemic. Several countries reported similar cases in 2019, but the specific origin of COVID-19 remained unidentified (1). Globally, by May 28, 2020, ~5.79 million people were diagnosed with COVID-19, and the mortality reached 357,432 (2). Currently, COVID-19 is the most important health crisis worldwide. The latest COVID-19 research has focused on severely and critically ill patients because disease progression to this stage leads to rapid patient deterioration that can easily lead to inflammatory storm, respiratory distress, multiple organ dysfunction, and eventually, death. Therefore, the treatment of severe and critical cases is a priority. However, severe acute respiratory syndrome coronavirus 2 (SARS-CoV-2), which causes COVID-19, is highly transmissible; the available medical treatment modality is limited, with no standard curative modality identified to date. Thus, the treatment of severe COVID-19 has been extremely difficult (3, 4). Accordingly, it is important to explore early predictors of disease progression to provide timely intervention. In addition to categorizing the patient's condition as mild, moderate, severe, and critical, the “COVID-19 Treatment and Diagnosis Guidelines (Seventh Trial Version)” added three laboratory indicators namely, reduced lymphocyte count, inflammatory factors, and lactic acid level (5). A single-center retrospective study conducted in Wuhan Union Hospital reported that the reduction in lymphocytes and the pro-inflammatory cytokine storm are related to disease severity (6). With increasing research on severe COVID-19, T lymphocytes, lactic acid, and interleukin-6 (IL-6) have been identified to be helpful in the early identification of severe disease (7–10).

Although early indicators for severe COVID-19 have already been reported, the patients' general condition as well as the admission and treatment criteria differ across countries. Further, most of these criteria and evaluation systems are based on data from Wuhan, China, where the outbreak was first reported. Therefore, the values of these indicators or evaluation systems require further verification.

This study aimed to identify early indicators of disease progression to provide the basis for improved prognostic prediction and disease management. We included cases from Shanghai and selected indicators representative of mild, moderate, severe, and critical disease at admission, for analysis. In addition, this study focused on the comparison of general information, clinical manifestations, past histories, and laboratory indicators between moderate cases for which the disease progressed to severe condition and mild and moderate cases that remained stable.

## Methods

### Research Design and Subjects

This study was approved by the Ethics Committee of Shanghai Public Health Clinical Center (SHPHC) (YJ-2020-S028-02). The need for informed consent was waived owing to the COVID-19 outbreak in 2019. Fifty-three COVID-19 patients who were admitted at and discharged after recovery from SHPHC between January 20, 2020, and February 20, 2020, were included. Diagnoses were confirmed according to the “COVID-19 and Diagnosis Guidelines of the National Health Commission of the People's Republic of China (Fifth Trial Edition)” and reviewed according to the “COVID-19 and Diagnosis Guideline of the National Health Commission of the People's Republic of China (Seventh Trial Edition).” All the patients were examined for consistent items immediately after admission. The patients were managed with the same program (the same laboratory tests and the same general principles of treatment were applied). Although the approach to management differs according to the individual situation of each patient, the management itself did not vary when compared with the clinical and laboratory manifestations. Patients whose data were mostly missing were excluded. A patient was categorized as having mild severity if the following criteria were met: mild disease was defined as mild clinical symptoms, but no pneumonia symptoms observed on imaging. Moderate disease was defined as the occurrence of fever and respiratory symptoms as well as pneumonia features observed on imaging. Severe disease was defined if one of the following criteria was met: (1) shortness of breath, with respiratory rate (RR) ≥30 beats/min; (2) blood oxygen saturation ≤ 93% under the resting state; (3) partial pressure of oxygen (PaO_2_)/fraction of inspired oxygen (FiO_2_) ≤ 300 mmHg; and (4) significant disease progression > 50% in lung images within 24–48 h. Finally, a patient was identified as having critical disease if, in addition to the above criteria, one of the following was met: (1) respiratory failure that requires mechanical ventilation; (2) shock; and (3) multiple organ failure that requires intensive care unit (ICU) monitoring.

### Data Collection

Data at admission, laboratory test results, and imaging examination results were collected from the electronic medical records and nursing records. The data were validated through direct inspection by the medical officers in charge, thereby ensuring data integrity. At admission, we collected data on age, sex, contact history, chronic disease history (hypertension, diabetes, malignancy, heart disease, lung disease, liver disease, kidney disease, and thyroid disease), and admission symptoms (fever, cough, sputum, chest tightness, shortness of breath, headache, myalgia, diarrhea, nausea, poor appetite, inappetence, and fatigue).

### Statistical Analysis

Data on age and time from onset to admission were reported as median (interquartile range), whereas other continuous variables were expressed as mean ± standard deviation (SD). Comparisons were conducted using the independent sample *t*-test, Fisher's exact test, and χ^2^-test. The primary study outcome was the identification of indicators with the highest early predictive value based on the survival curves. The survival curves were plotted from the optimal statistical time windows of mild and moderate cases that remained stable and moderate cases that progressed to severe conditions in MaxStat software. The secondary outcome was the identification of supportive indicators for disease progression after admission. We combined mild and moderate patients into one group and severe and critically ill patients into another group and analyzed the various laboratory tests between the two groups to explore the early warning indicators of COVID-19. Pairwise comparisons were subsequently conducted between data from mild and moderate cases and from severe and critical cases. Then, we used the same group (mild and moderate patients) mentioned above and analyzed the various laboratory tests between the patients of this group who progressed to severe and stable conditions. In addition, trends in continuous data of patients with mild and moderate conditions that remained stable and those with mild and moderate disease that progressed to severe conditions were analyzed. For all analyses, *P* < 0.05 was considered statistical significance.

## Results

### Patient Characteristics

Of the 53 patients, 26 had moderate disease at admission, and their conditions were stable during the treatment; 18 had moderate disease at admission, and the disease progressed to severe during the treatment; 9 had severe or critical disease at admission, and the condition did not improve. The patients were categorized into four groups according to their condition at admission: mild (*n* = 3), moderate (*n* = 41), severe (*n* = 7), and critical (*n* = 2). They were also categorized according to disease progression into those with mild or moderate conditions that remained stable (*n* = 26), those with moderate disease that progressed to severe conditions (*n* = 18), and those whose conditions were continuously severe or critical (*n* = 9). The patient characteristics are shown in Table 1. The median patients' age in the overall cohort was 60.5 (range, 41.3–67.5) years. Mild or moderately ill patients were significantly younger than severely or critically ill patients (median age: 54 [range, 40.5–64.5] vs. 70 [range, 60.0–75.0] years, *p* = 0.001). The majority of the patients were male (34 vs. 19). The most common symptom was fever (83.02%), followed by cough (37.74%) and sputum production (24.53%). In addition, compared with the mildly or moderately ill group, there were more patients in the severely and critically ill group who experienced shortness of breath (*p* = 0.005). However, there were no significant differences in other symptoms such as fever, cough, sputum, chest tightness, fatigue, and gastrointestinal discomfort between the two groups. Over 70% of the patients had underlying diseases including 5 (9.43%) with diabetes, 16 (30.19%) with hypertension, 7 (13.21%) with heart disease, and 2 (3.77%) with cancer. The proportions of patients with diabetes (*p* = 0.030) and heart disease (*p* = 0.012) were significantly higher in the severely and critically ill group than in the mild and moderately ill group.

**Table 1:** Baseline information of COVID-19 patients enrolled in this study.

	**All patients**	**Mild and moderate (*n* = 44)**	**Severe and critical (*n* = 9)**	*P-*value^*^
Age, Median (IQR)-yrs	60.5 (41.3–67.5)	54 (40.5–64.5)	70 (60.0–75.0)	0.0010^†^
Gender, *n* (%)				0.13
Male	34 (64.15)	26 (59.09)	8 (88.89)	
Female	19 (35.85)	18 (40.91)	1 (11.11)	
Exposure, *n* (%)				0.50
Wuhan-direct	26 (49.06)	21 (47.73)	5 (55.56)	
Wuhan-indirect	13 (24.53)	10 (22.73)	3 (33.33)	
No explicit contact	14 (26.42)	13 (29.55)	1 (11.11)	
Onset to admission, Median (IQR)-days	4 (3–7)	4 (3–7)	4 (3–10)	0.71
Main symptoms, *n* (%)				
No any symptoms	3 (5.66)	3 (6.82)	0 (0.00)	0.42
Fever	44 (83.02)	37 (84.09)	7 (77.78)	0.65
Cough	20 (37.74)	18 (40.91)	2 (22.22)	0.46
Headache	3 (5.66)	3 (6.82)	0 (0.00)	0.42
Myalgia	2 (3.77)	2 (4.55)	0 (0.00)	0.43
Sputum production	13 (24.53)	12 (27.27)	1 (11.11)	0.31
Diarrhea	1 (1.89)	1 (2.27)	0 (0.00)	0.65
Chest tightness	7 (13.21)	5 (11.36)	2 (22.22)	0.38
Anhelation	6 (11.32)	2 (4.55)	4 (44.44)	0.0050
Dyspnea	1 (1.89)	0(0.00)	1 (11.11)	0.17
Fatigue	9 (16.98)	7(15.91)	2 (22.22)	0.64
Nausea	2 (3.77)	2 (4.55)	0 (0.00)	0.51
Poor appetite	5 (9.43)	4 (9.09)	1 (11.11)	0.85
Underlying disease				
Hypertension	16 (30.19)	12 (27.27)	4 (44.44)	0.43
Diabetes	5 (9.43)	2 (4.55)	3 (33.33)	0.030
Liver disease	2 (3.77)	1 (2.27)	1 (11.11)	0.31
Lung disease	3 (5.66)	2 (4.55)	1 (11.11)	0.44
Heat disease	7 (13.21)	3 (6.82)	4 (44.44)	0.012
Thyroid disease	2 (3.77)	2 (4.55)	0 (0.00)	0.54
Kidney disease	1 (1.89)	0 (0.00)	1 (11.11)	0.17
Cancers	2 (3.77)	1 (2.27)	1 (11.11)	0.31

*Data are median (IQR), n (%), or n/N (%), where N is the total number of patients with available data. P-values compare patients with mild & moderate disease with patients with severe & critical disease using χ^2^-test, Fisher's exact test, or student t-test*.

* *χ^2^-test or Fisher's exact test*,

† *Student t-test*.

The severely and critically ill group showed significantly higher results in 16 laboratory indicators than the mild and moderately ill group including B-type natriuretic peptide precursor (*p* < 0.0001), C-reactive protein (CRP) (*p* = 0.0040), D-2 polymer (*p* = 0.0080), IgA (*p* = 0.0030), leukocyte count (*p* < 0.0001), procalcitonin (*p* = 0.015), thrombin time (*p* = 0.0010), lactate (*p* = 0.0030), lactate dehydrogenase (*p* = 0.0010), total calcium (*p* = 0.0090), and neutrophil count (*p* < 0.0001) (Table 2). In contrast, the severely and critically ill group showed significantly lower values than the mild and moderately ill group in 12 laboratory indicators including CD3 percentage (*p* = 0.0010), absolute CD4 count (*p* = 0.0070), albumin (*p* = 0.0070), and lymphocyte count (*p* = 0.049) (Table 2).

**Table 2:** Comparison of laboratory examination between patients with mild & moderate or severe & critical types disease.

	**Mild and moderate (*N* = 44)**	**Severe and critical (*N* = 9)**	
	**Mean ± SD**	**Mean ± SD**	*P*-value^*^
**Blood routine**			
Leukocyte count, × 10^9^ per L	4.64 ± 1.74	9.21 ± 6.23	<0.0001
Lymphocyte count, × 10^9^ per L	1.09 ± 0.74	0.58 ± 0.29	0.049
Lymphocyte, %	22.28 ± 8.90	9.47 ± 8.08	<0.0001
Neutrophil count, × 10^9^ per L	3.10 ± 1.36	8.09 ± 6.09	<0.0001
Neutrophil, %	67.59 ± 10.04	83.69 ± 11.23	<0.0001
Monocyte count, × 10^9^ per L	2.69 ± 11.41	0.49 ± 0.38	0.57
Monocyte, %	9.25 ± 4.77	6.21 ± 4.33	0.084
Eosinophil count, × 10^9^ per L	0.02 ± 0.07	0.03 ± 0.08	0.70
Eosinophil, %	0.23 ± 0.51	0.47 ± 1.10	0.31
**Blood biochemistry**			
Partial blood oxygen pressure, KPa	13.45 ± 4.86	14.32 ± 7.07	0.65
Hemoglobin, g/L	15.84 ± 3.37	15.12 ± 2.27	0.54
Standard bicarbonate, mmol/L	24.26 ± 1.61	22.59 ± 2.90	0.019
Residual alkali, mmol/L	−0.34 ± 2.28	−2.49 ± 3.60	0.026
Oxygen saturation, %	97.24 ± 1.92	95.94 ± 3.08	0.11
Carbon dioxide, mmol/L	21.01 ± 2.33	20.08 ± 2.11	0.27
Lactate, mmol/l	2.53 ± 0.78	3.46 ± 1.07	0.0030
Bilirubin (Arterial blood), μmol/L	9.47 ± 5.88	15.44 ± 9.90	0.018
L-γ-glutamyltransferase, U/L	46.60 ± 42.25	39.00 ± 36.34	0.62
Glutathione reductase, U/L	79.54 ± 17.63	101.90 ± 25.67	0.0020
Alkaline phosphatase, U/L	55.82 ± 22.01	57.44 ± 9.99	0.83
Lactate dehydrogenase, U/L	284.70 ± 109.62	425.33 ± 132.62	0.0010
Total bilirubin, μmol/L	9.32 ± 4.53	11.03 ± 5.28	0.32
Direct bilirubin, μmol/L	6.12 ± 10.85	6.34 ± 3.33	0.95
Total protein, g/L	68.43 ± 5.32	64.08 ± 6.22	0.035
Albumin, g/l	38.91 ± 5.40	33.31 ± 5.62	0.0070
A/G, %	1.63 ± 1.55	1.10 ± 0.26	0.32
Prealbumin, g/L	119.51 ± 45.66	77.44 ± 45.89	0.015
Apolipoprotein A, g/L	0.95 ± 0.19	0.78 ± 0.12	0.0090
Low density lipoprotein, mmol/L	2.80 ± 1.13	3.58 ± 1.30	0.072
High density lipoprotein, mmol/L	1.22 ± 0.48	0.89 ± 0.21	0.052
Glucose, mmol/L	7.07 ± 1.55	13.61 ± 8.93	<0.001
Uric acid, μmol/L	257.00 ± 98.52	330.02 ± 215.73	0.12
Haptoglobin, mg/dl	234.61 ± 99.92	280.61 ± 96.84	0.21
Acid glycoprotein, mg/dl	170.38 ± 44.73	187.69 ± 37.37	0.28
B-type natriuretic peptide precursor, pg/ml	106.25 ± 213.62	679.95 ± 653.75	<0.0001
Creatinine, μmol/L	70.18 ± 18.24	120.98 ± 111.87	0.006
Retinol binding protein, mg/L	22.53 ± 7.47	20.78 ± 9.77	0.55
Total calcium, mmol/L	2.01 ± 0.10	1.90 ± 0.12	0.0090
**Coagulation function**			
D-dimer, μg/ml	1.01 ± 2.98	4.89 ± 6.65	0.0080
INR	1.04 ± 0.18	1.06 ± 0.11	0.67
PTA, %	102.70 ± 19.55	93.11 ± 14.08	0.17
Activated partial thromboplastin time, s	41.98 ± 9.26	40.08 ± 6.37	0.56
Thrombin time, s	16.28 ± 1.10	18.13 ± 2.32	0.0010
Prothrombin time, s	13.42 ± 0.95	13.94 ± 1.15	0.15
Fibrinogen, g/l	4.61 ± 1.18	5.69 ± 1.75	0.027
**Immunology**			
C3, g/l	1.13 ± 0.25	1.13 ± 0.27	0.99
CD3+ lymphocyte, %	65.02 ± 13.88	48.20 ± 6.68	0.0010
CD3+ lymphocyte count, /μL	720.82 ± 691.59	274.00 ± 183.26	0.062
CD4+ lymphocyte, %	38.73 ± 11.11	27.72 ± 8.30	0.0070
CD4+ lymphocyte count, /μL	384.48 ± 173.96	163.11 ± 147.02	0.0010
CD8+ lymphocyte, %	23.35 ± 11.10	18.63 ± 6.78	0.23
CD8+ lymphocyte count, /μL	303.59 ± 598.73	102.78 ± 67.25	0.32
CD45+ lymphocyte count, /μL	1029.84 ± 753.77	545.67 ± 286.88	0.065
CD4/CD8	1.94 ± 0.82	1.76 ± 1.08	0.56
IgA, g/l	2.29 ± 0.93	3.57 ± 1.77	0.0030
IgG, g/l	12.06 ± 1.79	12.22 ± 2.66	0.83
IgM, g/l	1.06 ± 0.48	1.05 ± 0.44	0.96
**Infection-related biomarkers**			
C-reactive protein, mg/l	38.01 ± 41.03	94.60 ± 84.99	0.0040
Erythrocyte sedimentation rate, mm/h	64.77 ± 35.76	80.13 ± 28.77	0.26
Procalcitonin, ng/ml	0.14 ± 0.33	0.54 ± 0.79	0.015

*Laboratory indicators of mild & moderate or severe & critical patients with available data. P-values compare patients with mild & moderate disease with patients with severe & critical disease using independent sample student t-test*.

* *Independent sample student t-test*.

Older age patients, those with underlying disease including diabetes and heart disease, male patients, and those with significantly higher or lower values in one of the above laboratory indicators often experienced disease progression and had poor prognosis. This indicates that these factors may predict the progression to severe disease.

### Comparison Between Mild and Moderate Patients That Progressed to Severe Disease or Remained Stable

For further analysis of the early warning indicators of disease progression, we compared the prognosis among the 44 patients with mild or moderate disease at admission. Of these, 26 remained stable and were eventually cured and discharged. Further, 18 patients developed progression to severe disease. Analysis of the demographic characteristics, past histories, admission symptoms, and laboratory test results of these two groups showed that those with disease progression were significantly older (mean age, 64.0 [range, 49.5–69.5] vs. 49.0 [40.0–59.0] years, *p* = 0.045) and were higher in males (77.78 vs. 46.15%, *p* = 0.036) (Table 3). The remaining clinical manifestations (e.g., representative symptoms such as fever, cough, fatigue, and myalgia) and underlying diseases (e.g., diabetes, hypertension, and heart disease) were not significantly different between the two groups (Table 3).

**Table 3:** Baseline information of COVID-19 mild and moderate patients with or without progressive disease.

	**Stable disease (*n* = 26)**	**Progressive disease (*n* = 18)**	*P*-value^*^
Age, Median (IQR)-yrs	48.0 (40.0–59.0)	64.0 (49.5–69.5)	0.045^†^
Gender, *n* (%)			
Male	12 (46.15)	14 (77.78)	0.036
Female	14 (53.85)	4 (22.22)	
Exposure, *n* (%)			0.61
Wuhan-direct	14 (53.85)	7 (38.89)	
Wuhan-indirect	5 (19.23)	5 (27.78)	
No explicit contact	7 (26.92)	6 (33.33)	
Onset to admission, Median (IQR)-days	4.0 (3.0–6.5)	5.0 (3.0–6.8)	0.53
Main symptoms, *n* (%)			
No any symptoms	3 (11.54)	0 (0.00)	0.26
Fever	20 (76.92)	17 (94.44)	0.21
Cough	11 (42.31)	7 (38.89)	0.82
Headache	3 (11.54)	0 (0.00)	0.26
Myalgia	2 (7.69)	0 (0.00)	0.23
Sputum production	7 (26.92)	5 (27.78)	0.95
Diarrhea	1 (3.85)	0 (0.00)	0.40
Chest tightness	3 (11.54)	2 (11.11)	0.97
Anhelation	1 (3.85)	1 (5.56)	0.79
Fatigue	6 (23.08)	1 (5.56)	0.21
Nausea	2 (7.69)	0 (0.00)	0.23
Poor appetite	3 (11.54)	1 (5.56)	0.63
Underlying disease, *n* (%)			
Hypertension	5 (19.23)	7 (38.89)	0.18
Diabetes	0 (0.00)	2 (11.11)	0.16
Liver disease	0 (0.00)	1 (5.56)	0.41
Lung disease	0 (0.00)	2 (11.11)	0.16
Heat disease	1 (3.85)	2 (11.11)	0.35
Thyroid disease	1 (3.85)	1 (5.56)	0.79
Cancers	1 (3.85)	0 (0.00)	0.40

*Data are median (IQR), n (%), or n/N (%), where N is the total number of patients with available data. P-values compare mild & moderate patients with stable disease with patients with progressive disease using χ^2^-test, Fisher's exact test, or student t-test*.

* *χ^2^-test or Fisher's exact test*,

† *Student t-test*.

Compared with the stable group, the disease progression group had significantly lower CD3% (*p* = 0.032), CD4% (*p* = 0.0060), absolute CD4 lymphocyte count (*p* = 0.0040), and total calcium level (*p* = 0.0020) as well as significantly higher CRP (*p* = 0.0010) and lactate dehydrogenase (*p* = 0.0010) values, along with higher values of 5 other indicators (Table 4). Factors such as people of an old age (older), man, and significantly higher or lower value in one of the above laboratory indicators were associated with disease progression. The patients in the disease progression group also had blood coagulation as well as liver and kidney function disorders. These results indicate that these factors can be used as early indicators for disease progression in COVID-19 patients.

**Table 4:** Comparison of Laboratory examination between mild and moderate patients with or without progressive disease.

	**Stable disease (*n* = 26)**	**Progressive disease (*n* = 18)**	
	**Mean ± SD**	**Mean ± SD**	*P-*value^*^
**Blood routine**			
Leukocyte count, × 10^9^ per L	4.29 ± 1.33	5.16 ± 2.14	0.10
Lymphocyte count, × 10^9^ per L	1.09 ± 0.30	1.08 ± 1.13	0.95
Lymphocyte, %	24.03 ± 7.42	19.74 ± 10.38	0.12
Neutrophil count, × 10^9^ per L	2.75 ± 1.08	3.60 ± 1.58	0.040
Neutrophil, %	65.19 ± 7.23	71.06 ± 12.52	0.056
Monocyte count, × 10^9^ per L	4.24 ± 14.75	0.46 ± 0.43	0.29
Monocyte, %	9.68 ± 3.73	8.62 ± 6.04	0.47
Eosinophil count, × 10^9^ per L	0.03 ± 0.08	0.01 ± 0.06	0.50
Eosinophil, %	0.28 ± 0.42	0.14 ± 0.61	0.37
**Blood biochemistry**			
Partial blood oxygen pressure, KPa	13.27 ± 4.54	13.72 ± 5.44	0.77
Hemoglobin, g/L	15.28 ± 2.75	16.70 ± 4.07	0.18
Standard bicarbonate, mmol/L	24.54 ± 1.30	23.82 ± 1.96	0.15
Residual alkali, mmol/L	0.15 ± 1.76	−1.05 ± 2.76	0.096
Oxygen saturation, %	97.45 ± 1.78	96.92 ± 2.13	0.38
Carbon dioxide, mmol/L	21.55 ± 1.66	20.19 ± 2.96	0.060
Lactate, mmol/l	2.49 ± 0.89	2.58 ± 0.58	0.71
Bilirubin (Arterial blood), μmol/L	8.44 ± 5.68	11.06 ± 5.99	0.16
L-γ-glutamyltransferase, U/L	44.15 ± 45.12	50.35 ± 38.46	0.64
Glutathione reductase, U/L	75.18 ± 14.57	86.21 ± 20.15	0.043
Alkaline phosphatase, U/L	56.00 ± 26.43	55.53 ± 13.42	0.95
Lactate dehydrogenase, U/L	240.42 ± 87.77	352.41 ± 107.00	0.0010
Total bilirubin, μmol/L	8.22 ± 3.24	11.01 ± 5.70	0.047
Direct bilirubin, μmol/L	6.67 ± 13.95	5.28 ± 1.89	0.69
Total protein, g/L	69.68 ± 4.67	66.50 ± 5.80	0.054
Albumin, g/l	40.20 ± 3.02	36.93 ± 7.45	0.051
A/G, %	1.78 ± 2.01	1.40 ± 0.14	0.44
Prealbumin, g/L	129.84 ± 45.69	103.72 ± 42.09	0.066
Apolipoprotein A, g/L	0.99 ± 0.20	0.89 ± 0.15	0.069
Low density lipoprotein, mmol/L	3.04 ± 1.28	2.45 ± 0.76	0.097
High density lipoprotein, mmol/L	1.29 ± 0.59	1.11 ± 0.21	0.23
Glucose, mmol/L	7.03 ± 1.69	7.14 ± 1.36	0.83
Uric acid, μmol/L	244.02 ± 63.12	276.85 ± 136.25	0.29
Haptoglobin, mg/dl	235.78 ± 89.23	232.81 ± 117.31	0.93
Acid glycoprotein, mg/dl	160.65 ± 45.59	185.27 ± 40.17	0.077
B-type natriuretic peptide precursor, pg/ml	63.04 ± 68.42	172.34 ± 323.73	0.10
Creatinine, μmol/L	63.44 ± 14.05	80.48 ± 19.44	0.0020
Retinol binding protein, mg/L	23.53 ± 7.47	21.01 ± 7.44	0.29
Total calcium, mmol/L	2.04 ± 0.09	1.95 ± 0.10	0.0020
**Coagulation function**			
D-dimer, μg/ml	0.52 ± 0.29	1.76 ± 4.72	0.19
INR	1.03 ± 0.20	1.04 ± 0.13	0.89
PTA, %	106.38 ± 21.45	97.06 ± 15.12	0.13
Activated partial thromboplastin time, s	39.68 ± 4.65	45.51 ± 13.02	0.042
Thrombin time, s	16.15 ± 0.81	16.47 ± 1.44	0.35
Prothrombin time, s	13.23 ± 0.54	13.72 ± 1.32	0.10
Fibrinogen, g/l	4.76 ± 1.20	4.38 ± 1.15	0.31
**Immunology**			
C3, g/l	1.17 ± 0.22	1.07 ± 0.27	0.19
CD3+ lymphocyte, %	68.72 ± 9.82	59.68 ± 17.16	0.032
CD3+ lymphocyte count, /μL	720.85 ± 213.42	720.78 ± 1069.03	1.0
CD4+ lymphocyte, %	42.42 ± 8.98	33.39 ± 11.95	0.0060
CD4+ lymphocyte count, /μL	445.50 ± 157.75	296.33 ± 161.34	0.0040
CD8+ lymphocyte, %	22.70 ± 6.84	24.29 ± 15.52	0.65
CD8+ lymphocyte count, /μL	238.85 ± 93.88	397.11 ± 937.07	0.40
CD4/CD8	2.04 ± 0.70	1.81 ± 0.97	0.37
CD45+ lymphocyte count, /μL	1025.35 ± 310.78	1036.33 ± 1137.98	0.96
IgA, g/l	2.40 ± 1.02	2.12 ± 0.78	0.35
IgG, g/l	12.29 ± 1.77	11.72 ± 1.82	0.31
IgM, g/l	1.10 ± 0.47	1.00 ± 0.50	0.50
**Infection-related biomarkers**			
C-reactive protein, mg/l	21.64 ± 25.28	62.57 ± 48.21	0.0010
Erythrocyte sedimentation rate, mm/h	68.38 ± 38.08	59.56 ± 32.46	0.43
Procalcitonin, ng/ml	0.07 ± 0.13	0.26 ± 0.48	0.063

*Laboratory indicators of mild or moderate conditions that remained stable patients or moderate disease that progressed to severe condition patients with available data. P-values compare mild & moderate patients with stable disease with patients with progressive disease using independent sample student t-test*.

* *Independent sample student t-test*.

### Indicators for Disease Progression

**Figure 1:**
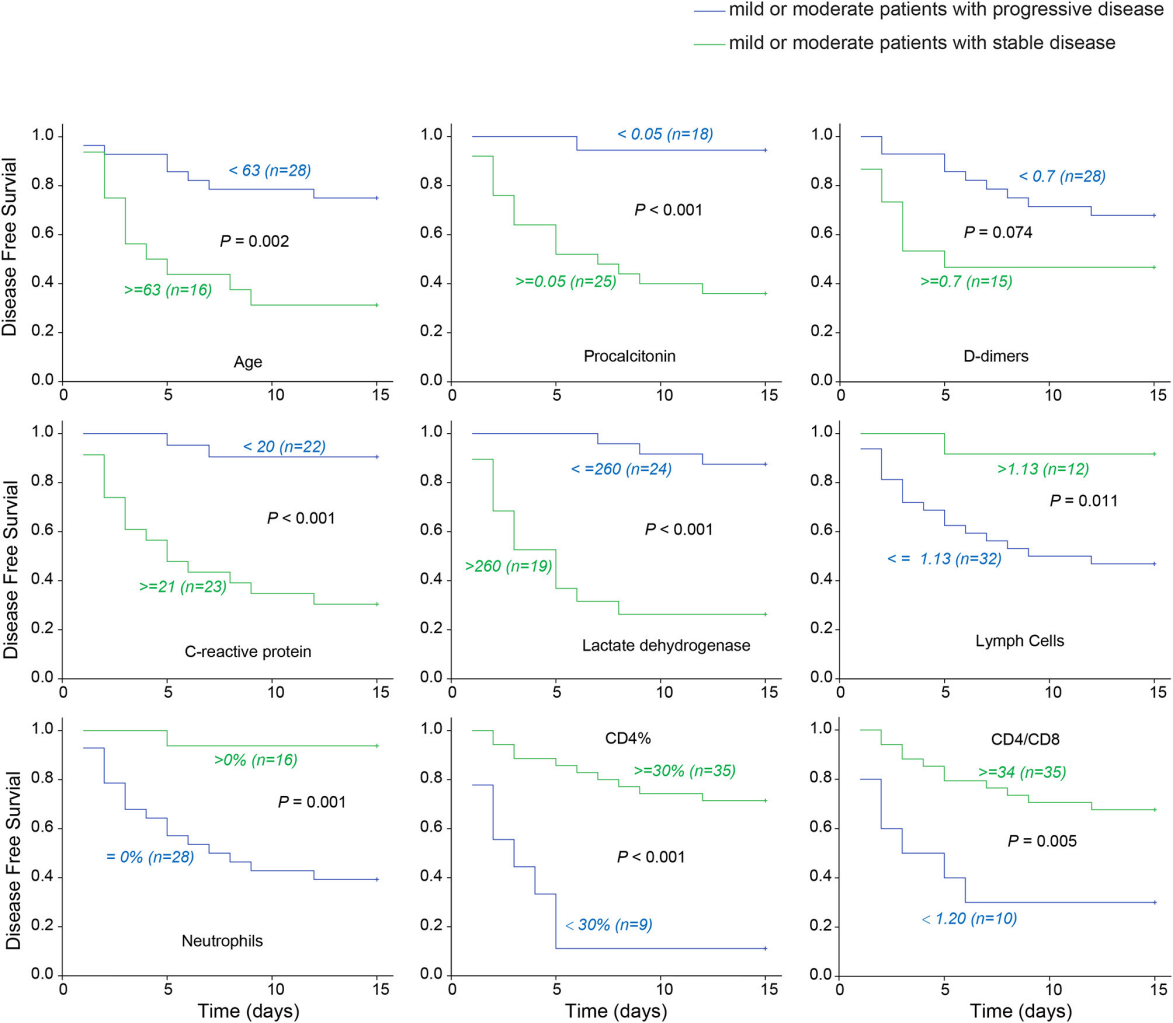
COVID-19 early warning indicator survival curve. The associations between severe disease free survival and early indicators in 43 patients with mild or moderate disease upon admission. Taking hospitalization to become severe as the end, the latest turn weight is 12 days, and the longest stable condition is 15 days. The optimal cut-points are selected for discriminating the patients to high or low risk who will develop severe disease using Maxstat software. Lactate dehydrogenase and procalcitonin etc. are early warning indicators to severe COVID-19.

With hospital admission as the starting point, and disease progression to severe condition as the end point, the longest duration to disease progression was 12 days, whereas the longest duration to stable condition was 15 days. To investigate the correlation between indicators and disease progression in COVID-19, survival curves were plotted in the optimal statistical time windows (maximally selected rank statistics with several *p*-value approximations) (11) of mild and moderate cases whose conditions remained stable, and moderate cases that progressed to severe conditions using MaxStat software. In total, 9 significant early indicators were identified including age (>64 years), procalcitonin, D-dimer, serum CRP, lactate dehydrogenase, lymphocytes, neutrophils, CD4%, and CD4/CD8 ratio (Figure 1). These factors were indicative of disease initiation and progression to severe COVID-19 during the early stage of the disease.

## Discussion

Currently, the incidence and mortality rates of COVID-19 have exceeded those of severe acute respiratory syndrome (SARS) in 2003 (8,273 confirmed cases and 775 deaths) and the Middle East respiratory syndrome (1,139 confirmed cases and 431 deaths). COVID-19 is also more easily transmissible than these two illnesses (12, 13). Although most COVID-19 patients only exhibit mild and moderate symptoms, some patients develop severe disease, which can lead to death. Patient management is further complicated by challenges in the treatment of severe COVID-19 cases due to complicated patient conditions, restricted treatment environment, and the absence of a specific curative strategy. Therefore, early indicators of disease initiation and progression to severe condition would be crucial for reducing the morality rate and improving prognosis.

Research on COVID-19 has shown that elderly male patients with comorbidities are at the highest risk of infection (8). Similarly, we found that people of an old age and man predict progression in patients. In addition, while elderly COVID-19 patients with underlying illness such as cerebrovascular disease, liver disease, kidney disease, or malignancy often die owing to their original comorbidities (3, 14), we found that the presence of underlying disease itself was a risk factor for progression to severe COVID-19. While a previous study showed that patients with hypertension have a higher risk of COVID-19 (10), we found no evidence to indicate that hypertension can be an early indicator of COVID-19. This finding may be owing to our small sample size, and the relationship between hypertension and COVID-19 needs to be further explored. Although hypertension was the most common underlying disease in the included patients, there was no significant difference in the rate of hypertension according to prognosis. Further, patients with diabetes and heart disease were more likely to become severely ill, which could possibly be due to an immunocompromised state and metabolic dysfunction.

The clinical manifestations of COVID-19 are viral pneumonia symptoms including fever, fatigue, dry cough, and diarrhea. Fever is the most common clinical manifestation of COVID-19, followed by cough (15, 16). In addition, chest tightness and shortness of breath are also important symptoms. In this study, the proportion of severe and critical cases with shortness of breath at admission was significantly higher than that of mild and moderate cases. Furthermore, analysis of the symptoms of all severe and critical cases in this study suggested that shortness of breath was often accompanied by accelerated disease progression in lung images. Several laboratory indicators have also been proposed as early indicators of severe COVID-19 (17, 18). For example, it has been reported that IL-6 and lactic acid can independently predict the progression of COVID-19 (10). Further, increased CRP and progressive decrease in the absolute lymphocyte count have also been observed in severe COVID-19 patients (19–21). Therefore, these factors have been incorporated in the “COVID-19 Diagnosis and Treatment Guidelines (Seventh Trial Version)” issued by the National Health Commission of the People's Republic of China as early indicators of severe COVID-19 cases. In line with these findings, our results showed that increased CRP and decline of absolute lymphocyte count occurred in severe COVID-19 patients. We also found a significant difference in lactic acid index between the severely ill group and the mild or moderately ill group. This result indicates that lactic acid could be an early indicator of disease progression. In addition, we found that lactate dehydrogenase showed significant difference in both comparisons. This was later verified in the survival curve analysis. An elevated lactate dehydrogenase level may reduce the effectiveness of lactic acid as an early indicator of COVID-19. As elevated lactate dehydrogenase activity indicates early myocardial injury, it has been adopted as an indicator for acute myocardial infarction (22). High lactate dehydrogenase levels are also associated with tissue injury occurring in various diseases, including pulmonary disorders such as pneumonia, and liver and kidney dysfunctions; therefore, corresponding treatments should be taken timeously to prevent further deterioration of the disease (23–25). Similarly, as COVID-19 can also cause pneumonia as well as heart, liver, kidney, and other organ dysfunctions, the patients may die from heart failure, shock, acute respiratory distress syndrome, arrhythmia, or renal failure (16). Therefore, lactate dehydrogenase plays a vital role as an early indicator of disease initiation and progression to severe disease. Yan et al. also confirmed lactate dehydrogenase and other indicators as crucial predictive biomarkers of disease mortality (24).

Studies have reported that the severity of pulmonary infection and immune injury in all patients with SARS 2003 was associated with the infiltration of large numbers of neutrophils and macrophages in the lungs. Similar events were observed in patients with COVID-19 (26, 27). After the onset of COVID-19, CD4+T lymphocytes are immediately activated and become pathogenic type 1 T helper (Th1) cells that produce granulocyte-macrophage colony-stimulating factor, and accelerate the inflammatory response (28). In our study, we found that the absolute count and percentage of CD4+T lymphocytes were significantly lower in the disease progression group than in the stable group. In addition, survival curve analysis indicated that the reduction of CD4/CD8 ratio was closely related to the development of disease. Procalcitonin, a calcitonin-related gene product expressed in human epithelial cells in response to bacterial infections, was substantially increased in this study. Procalcitonin levels increase within 6–12 h of infection in response to pro-inflammatory mediator release after bacterial invasion and correlate with disease severity and clinical outcome, in patients with infection (29); it can also indicate the occurrence of sepsis (30). Procalcitonin is more specific for bacterial infections than CRP or white cell count (29, 31). Its elevation indicates the appearance of secondary infection in COVID-19 patients, which is an important warning signal, indicating that patients have low immune function and that the disease has reached the progressive stage. Significant decreases or increases in the above indicators are possibly associated with immune function disorders during disease progression.

We also found that an abnormal coagulation function indicator was associated with disease initiation and progression in severe COVID-19 cases. Aside from a substantially higher D-dimer level, other indicators, including the thrombin time, prothrombin time, and fibrinogen content were also higher in these cases than in the mild and moderate cases. Concurrently, the survival curve analysis indicated that the D-dimer level was higher in the disease progression group than in the stable group. Overall, these findings indicate that severely ill COVID-19 patients developed blood coagulation function disorders and were at a certain stage of coagulation or bleeding. Other studies reported similar conclusions that COVID-19 patients in the ICU demonstrate higher prothrombin time and D-dimer level at admission, and their median D-dimer level is higher than that of non-ICU patients (14, 20). In addition, a recent autopsy of a patient who died of COVID-19 revealed extensive bleeding and abnormal thrombosis in the lung tissue (32). These results indicate that coagulation indicators can predict disease initiation and progression in severe COVID-19 cases.

This study has some limitations. First, although we included a total of 177 confirmed cases from January 20 to February 20, 2020, the COVID-19 outbreak was declared a Public Health Emergency of International Concern, with incomplete detection indicators in some cases while some patients were still at the hospitalization stage and remained under intensive care. Therefore, we included only 53 patients with relatively complete and representative data in the analysis, including all basic information and laboratory indicators of all types of patients, including all 27 patients with severe and critical disease. Second, this was a retrospective study. Although our data can facilitate early diagnosis and prognostic prediction of severe COVID-19, the findings need to be further verified.

In conclusion, age and laboratory indicators, such as lactate dehydrogenase, procalcitonin, and D-dimer, are early predictors of severe COVID-19. Shortness of breath at admission, past histories of diabetes and heart disease, and abnormalities in the 28 indicators, such as CD4 percentage and CRP, indicate that the patient is already severely ill or has a significant risk of progressing to severe conditions. Meanwhile, abnormalities in 11 indicators, such as CD4 percentage after admission, are risk factors for progression to severe condition. Moreover, coagulation function disorder is also an early indicator of the disease.

## Data Availability Statement

The raw data supporting the conclusions of this article will be made available by the authors, without undue reservation.

## Ethics Statement

This study was approved by the Ethics Committee of SHPHC (YJ-2020-S028-02). The need for informed consent was waived due to the COVID-19 outbreak in 2019.

## Author Contributions

YL, SW, WC, YS, and JW designed the study, take responsibility for the integrity of the data, and the accuracy of data analysis. YL, SW, WC, and KS were responsible for conducting the study protocol and collecting all pertinent data. KS, SG, JW, AL, and XR performed the statistical analysis and drafted the manuscript. SW, WC, and TW revised the final manuscript. All authors contributed to the article and approved the submitted version.

## Conflict of Interest

The authors declare that the research was conducted in the absence of any commercial or financial relationships that could be construed as a potential conflict of interest. The reviewer TC declared an affiliation with no collaboration, with several of the authors, YL, KS, YS, and WC, to the handling editor at the time of review.
